# Anticonvulsant Effect of Diazoxide against Dichlorvos-Induced Seizures in Mice

**DOI:** 10.1155/2013/697305

**Published:** 2013-12-17

**Authors:** Amin Jazayeri, Samira Zolfaghari, Sattar Ostadhadi

**Affiliations:** ^1^Department of Pharmacology, Faculty of Veterinary Medicine, Shahrekord University, Shahrekord 88514, Iran; ^2^Department of Biology, Faculty of Sciences, Tehran University, Tehran 14174, Iran; ^3^Department of Pharmacology, Faculty of Medicine, Tehran University of Medical Sciences, Tehran 14174, Iran

## Abstract

Dichlorvos, a synthetic organophosphate toxin, is used as pesticides. These toxins can be used as pesticides in farming and medicine for the devastation and/or elimination of ectoparasites of animals. Reports have shown that Dichlorvos generate seizure effects in various animals. Potassium channel opener is extensively used for medication of cardiovascular and other diseases. Studies have shown that potassium channel opener has anticonvulsant effects in different animal models. The goal of this study was to evaluate the effect of dizoxide on Dichlorvos-induced seizures in mice. In this research, the animals received different doses of Diazoxide (1, 2.5, 5, 10, and 20 mg/kg b.wt.) intraperitoneally 30 min before intraperitoneal injection of Dichlorvos (50 mg/kg b.w.t). After Dichlorvos injection, latency of clones, severity of seizure, and finally death as the fate were investigated. Results showed that Diazoxide dose-dependently decreased the severity of Dichlorvos-induced seizures, so that Diazoxide at a dose of 5 mg (the lowest, *P* < 0.05) and 20 mg/kg b.wt. (the highest, *P* < 0.001) has anticonvulsant effects. Thus, our data suggest that diazoxide as ATP-sensitive potassium channels opener has anticonvulsant activity against dichlorvas-induced seizure.

## 1. Introduction

Epilepsy is a chronic, recurrent, frequently progressive neurological disorder that affects 1-2% of the population worldwide [1]. It has been shown that epileptic seizure results from abnormal excessive or hypersynchronous neuronal discharges in brain [2]. Opening of plasmalemmal K^+^ channels leads to cellular hyperpolarization and, in excitable tissues possessing K_ATP_ channels, triggering the opening of such channels prevents excitation [3]. One potential antiepileptic mechanism that has not yet been developed is K^+^ channel opening. K^+^ channels play an important role in the control of all features of neuronal excitability, such as resting membrane potential, responsiveness to synaptic inputs, spike frequency adaptation, and neurotransmitter release [4]. The therapeutic potential uses of K^+^ channel openers in the cardiovascular area (as antihypertensives and, in particular, as anti-ischemic agents in heart and skeletal muscle) and in asthma (where they reverse determined airway hyperreactivity) will also be examined [3]. During seizures extracellular pottasium concentration amplified, while intracellular pottasium concentration decreased [5]. On the other hand, pharmacological reports have shown that K_ATP_ channels play an important role in the management of seizure threshold in several in vitro and in vivo models [6, 7].

Organophosphorus agents are usually esters, amides, or thiol derivatives of phosphonic acid. They form a large family of ~50 000 chemical compounds with biological effects that have imperative and sometimes unique implications for man [8]. Organophosphorus (OP) compounds are cholinesterase-inhibiting chemicals used as pesticide [9]. Exposures to OPs are source of significant quantity of poisonings that affect various organs such as skeletal muscles, GI tract, bladder, secretory glands, CNS, and respiratory systems and create many signs and symptoms such as weakness, glandular secretion, fasciculation, acute pancreatitis, convulsion, respiratory depression, and finally death [10, 11]. Centrally mediated seizures and convulsions are one of the toxic signs that happen following poisoning with organophosphorus (OP) anticholinesterase such as Dichlorvos [12, 13]. Since treatment of this life-threatening effect of OP compounds is very importants so the aim of this study was to determine the effect of diazoxide (potassium channel opener) on Dichlorvos-induced seizures in mice.

## 2. Materials and Methods

*Animals*. Male mice NMRI weighing 20–26 g were used in the research. The animals were kept in a room with controlled temperature (21-22°C) and light (12 h light-dark cycle). The animals were permitted free access to standard laboratory food and tap water. All procedures were carried out in accordance with institutional guidelines for animal care and use. Assignment of animals to experimental groups (*n* = 8–10) was randomized. The tests were performed between 09:00 and 13:00 h. Dichlorvos solved in Tween 80 (5%) and Dioxide was dissolved in methanol. Animals were divided randomly and placed in treatment groups (*n* = 10). First, seizures were assessed in animals receiving Dichlorvos and then the effect of Tween 80 on seizures as control groups was evaluated. In other groups, different doses of Diazoxide (2.5, 5, 10, and 20 mg/kg) were injected 30 min before the intraperitoneal injection of Dichlorvos (50 mg/kg b.w.). After intraperitoneal injection of Dichlorvos animals were monitored by video camera for 120 minutes. Seizures were evaluated according to the subsequent qualitative staging system defined by McLean et al. [14]: Stage 0, no abnormal behavior; Stage 1, excessive salivation, chewing, and pawing of whiskers and mouth; Stage 2, dazed appearance, intermittent motionlessness, tremor, and/or bobbing of the head; Stage 3, like Stage 2, with random and/or generalized jerks; Stage 4, intermittent rearing on hind legs with forepaws extended (with clonic jerking) without falling; Stage 5, like Stage 4, with falling to the side or rear; Stage 6, status epilepticus. Stages 1–3 and 4–6 were considered as subconvulsive and convulsive behaviors, respectively. The latency of clonic seizures after injection of Dichlorvos (second), the latency to onset of death within one hour (second), mortality after injection of Dichlorvos (percentage), and stage of seizures induced by injection of Dichlorvos (percentage) were recorded. After testing data as the mean ± SEM expression and to analyze data, ANOVA followed by Tukey multiple comparison tests were used. The value of *P* < 0.05 to determine significance between groups was considered.

## 3. Result

**Figure 1:**
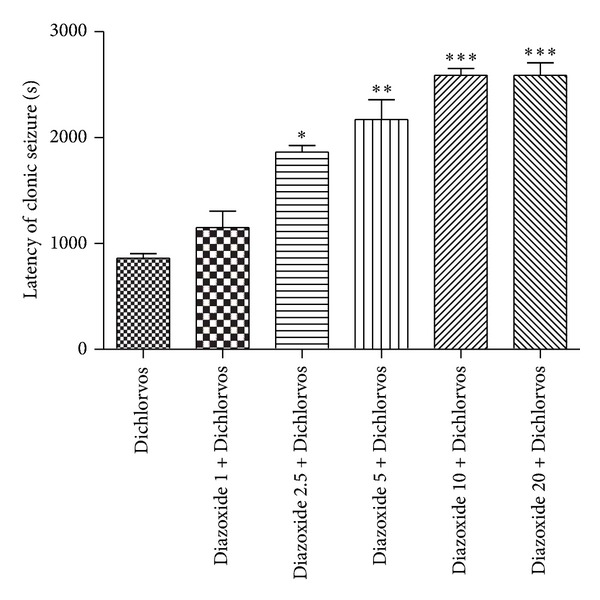
Effect of different doses of Diazoxide (mg/kg) on the starting time of clonic seizures after injection of Dichlorvos (50 mg/kg) (second). Data are shown as mean ± SEM. *P < 0.05, **P < 0.01, and ***P < 0.001 compared Dichlorvos group. (*n* = 10).

**Figure 2:**
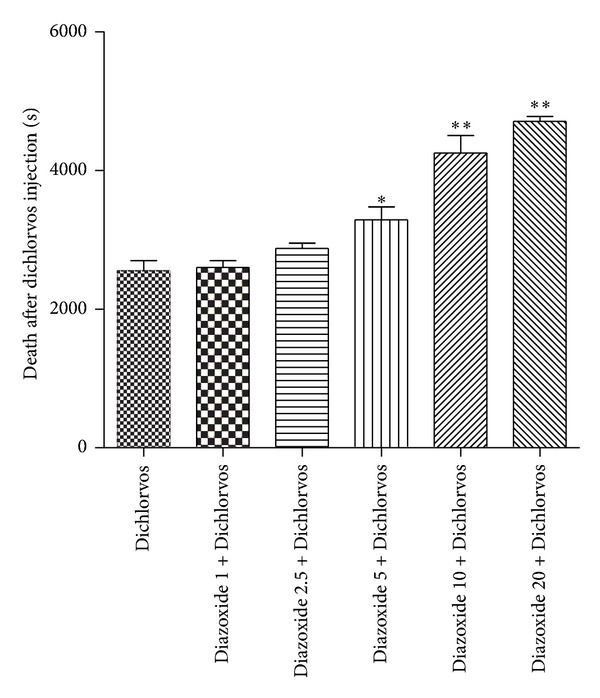
Effect of different doses of Diazoxide (mg/kg) on generation time of death after Dichlorvos injection (50 mg/kg) (second). Data are shown as mean ± SEM. *P < 0.05 and **P < 0.001 compared with Dichlorvos group. (*n* = 10).

**Figure 3:**
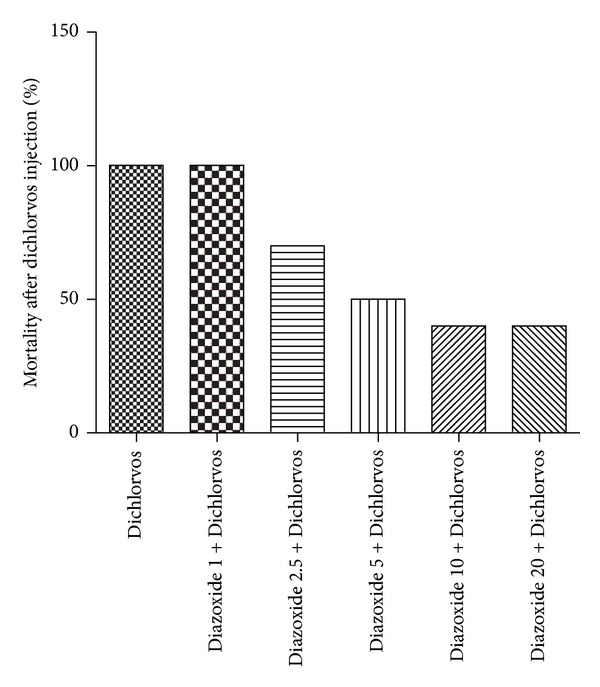
Effect of different doses of Diazoxide (mg/kg) on the mortality percentage after injection of Dichlorvos (50 mg/kg) (*n* = 10).

The effect of Tween 80 as a vehicle on Dichlorvos-induced seizures presented that this agent has no significant effect on seizures. Therefore, the results had not been displayed in graphs and tables. Effect of different doses of Diazoxide (1, 2.5, 5, 10, and 20 mg kg) on Dichlorvos-induced seizures showed that this drug dose-dependently reduced seizures. Pretreatment with Dioxide at 1 mg/kg did not prevent the Dichlorvos-induced behavioral seizures in mice (Figures 1 and 2 and Table 1). Diazoxide at doses 2.5 mg/kg (*P* ≤ 0.05), 5 mg/kg (*P* ≤ 0.01), 10, and 20 mg/kg (*P* ≤ 0.001) increased latency of clonic seizure after Dichlorvos injection (Figure 1). Also the time of death after Dichlorvos injection was increased by Diazoxide pretreatment at doses 5 mg/kg (*P* ≤ 0.05), 10, and 20 mg/kg (*P* ≤ 0.001) (Figure 2). Concomitant preinjection with Diazoxide lowered the lethality of Dichlorvos in mice, compared to Dichlorvos animals (Figure 3). So the most anticonvulsant effect of Diazoxide on the mortality and severity of seizures with a dose of 10 and 20 mg kg^1^ was observed.

## 4. Discussion

Dichlorvos cause clonic and tonic seizures and ultimately death. After the mice received intraperitoneal Dichlorvos, some degree of tremor and excessive activity showed that over time the symptoms became more severe and cause death. Diazoxide dose-dependently reduced clonic and tonic seizures and time of death after Dichlorvos.

Seizures and convulsions are one of the toxic effect that happen following poisoning with organophosphorus (OP) anticholinesterase such as Dichlorvos [15]. Depending on the level of AChE inhibition, cholinergic motivation may lead to hyperactivity of excitable tissues, causing fasciculations, seizures, convulsions, severe muscle paralysis, hypersecretion from secretory glands, respiratory failure, coma, and death [16]. Seizures, convulsions, and CNS lesions are distinctive results of systemic application of sublethal doses of AChEIs [17]. Drugs typically used against epilepsy in hospital are ineffective against organophosphate intoxication [18].

Potassium (K^+^) channels are the largest family of ion channels. Among the different kinds of  K^+^ channels, ATP-sensitive K^+^ (K_ATP_) channels are contributed in numerous physiological functions [19]. K_ATP_ channels are located pre- and postsynaptically in many brain areas and their works are controlled by the metabolic condition of the neuron. They open and close in reply to alterations in intracellular ATP/ADP relations. Low ATP degree open these channels, letting K^+^ efflux and cell hyperpolarization [20]. Pharmacological experiments have shown that K_ATP_ channels play a significant role in the regulation of seizure threshold in several in vitro and in vivo models [6, 7, 21]. K_ATP_ channels opener has been displayed to decrease excitability in CA3 hippocampal cells [22] and to show antiepileptic effects in a model of drug-induced epilepsia [23]. Molecular studies have shown that functional K_ATP_ channels are octameric complexes containing four inward rectifier K^+^ channel subunits (Kir6.1 or Kir6.2) and four sulfonylurea receptor subunits (SUR1, SUR2A, or SUR 2B), with diverse neurons expressing special combinations of K_ATP_ subunits [4]. Mice with lacks in expression of either the SUR1 gene or the Kir6.1 gene are susceptible to kainic acid-induced seizures, an animal model of human temporal lobe epilepsy [24]. Soundarapandian et al. have also established that expression of functional Kir6.1/SUR1 channels can be reflected as an endogenous cellular defensive event against epileptic activity via diminishing the danger of overactivation of glutamate transmission at CA3 synapses [25]. Moreover, it has been shown that mutant mice lacking the Kir6.2 subunit of K_ATP_ channels [knockout (KO) mice] were disposed to generalized seizures after brief hypoxia [7]. Transgenic mice, overexpressing the SUR1 gene in the forebrain, display a substantial increase in the threshold for kainate-induced seizures [26]. Also, it was recently reported that K_ATP_ channel openers such as cromakalim and Diazoxide increased the clonic seizures induced by PTZ in mice [27, 28].

## 5. Conclusion

In summary, this study showed that Diazoxide (K_ATP_ channel opener) decreasing clonic and tonic seizures from Dichlorvos in mice is probably the main mechanism which is anticonvulsant related to open potassium channel and increases potassium flow within neurons. So, further investigation is needed to evaluate the efficacy of this agent in AChE inhibition-induced seizure.

## Figures and Tables

**Table 1:** Effect of different doses of Diazoxide (mg/kg) on the various stages of seizures induced by Dichlorvos injection in mic (data are shown as percentage of mice, *n* = 10). Stage 0: no abnormal behavior; Stage 1: excessive salivation, chewing, and pawing of whiskers and mouth; Stage 2: dazed appearance, intermittent motionlessness, tremor, and/or bobbing of the head; Stage 3: like Stage 2, with random and/or generalized jerks; Stage 4: intermittent rearing on hind legs with forepaws extended (with clonic jerking) without falling; Stage 5: like Stage 4, with falling to the side or rear; Stage 6: status epilepticus.

Treatment	Stage of seizures		
	Stage 0	Stage 1–3	Stage 4–6
Dichlorvos	0	0	100
Diazoxide 1 + Dichlorvos	0	0	100
Diazoxide 2.5 + Dichlorvos	10	20	70
Diazoxide 5 + Dichlorvos	30	20	50
Diazoxide 10 + Dichlorvos	60	10	30
Diazoxide 20 + Dichlorvos	80	10	10
